# Growth and Neurodevelopmental Outcomes of Preterm Infants Born < 26 Weeks Gestation before and after Implementation of a Nutrition-Care Bundle

**DOI:** 10.3390/children11040475

**Published:** 2024-04-15

**Authors:** Giulia Res, Rosine F. Bishara, Paige Terrien Church, Rena Rosenthal, Rita Maria Bishara, Annie Dupuis, Elizabeth Asztalos, Rudaina Banihani

**Affiliations:** 1 Department of Newborn and Developmental Paediatrics, Sunnybrook Health Sciences Centre, 2075 Bayview Avenue, Toronto, ON M4N 3M5, Canada; giulia.res@sinaihealth.ca (G.R.); rosine.bishara@sunnybrook.ca (R.F.B.); rena.rosenthal@sunnybrook.ca (R.R.); ritabish@my.yorku.ca (R.M.B.); elizabeth.asztalos@sunnybrook.ca (E.A.); 2 Department of Paediatrics, University of Toronto, 555 University Avenue, Toronto, ON M5G 1X8, Canada; 3 Department of Pediatrics, Harvard Medical School, 25 Shattuck Street, Boston, MA 02115, USA; pchurch1@bidmc.harvard.edu; 4 Department of Neonatology, Beth Israel Deaconess Medical Center, 330 Brookline Avenue, Boston, MA 02215, USA; 5 Biostatistics Division, Dalla Lana School of Public Health, University of Toronto, 155 College Street, Toronto, ON M5T 3M7, Canada; annie.dupuis@mdstats.ca

**Keywords:** growth, neurodevelopmental outcomes, micro-preterm, nutrition bundle

## Abstract

Background: This study aimed to assess the impact of a nutrition-care bundle on growth and neurodevelopmental outcomes of micro-preterm infants born in a level III neonatal intensive care unit (NICU) by two years corrected age. Methods: A nutrition-care bundle emphasizing the prompt initiation of parenteral nutrition at birth, initiation of enteral feeds within 6 h after birth, and early addition of human milk fortifiers was implemented in 2015 for infants born < 26 weeks gestation. This before-and-after study evaluated growth and neurodevelopmental outcomes in infants born between 2012–2013 (before-nutrition-bundle, BNB) and 2016–2017 (after-nutrition-bundle, ANB). Results: A total of 145 infants were included in the study. Infants in the ANB group (n = 73) were smaller (birthweight and gestational age), and there were more male infants and multiples included compared to the BNB group (n = 72). Enteral feeds and fortifiers started earlier in the ANB group. Growth velocity and weight z-score changes were similar in both groups during NICU stay and post-discharge. Systemic steroid use, but not cohort, was linked to lower Bayley scores across all domains. Conclusions: Implementing a nutrition-care bundle was not consistently associated with improved weight gain and neurodevelopmental outcomes in the micro-preterm infant population, possibly due to ongoing high-quality nutritional care by the clinical team.

## 1. Introduction

Optimizing postnatal nutrition and growth in preterm infants is critical to mimic intrauterine nutrient retention, growth, and body composition while fostering functional development and mitigating potential outcome challenges [1,2,3]. In the first few days of life, preterm infants undergo contraction of the extracellular fluid compartment as they adapt to the extra-uterine environment, resulting in a weight z-score change of approximately −0.8 [3,4,5]. However, beyond this initial adjustment, various factors may aggravate early weight loss and hinder growth over time. These factors include prenatal influences, nutritional practices such as delayed initiation and advancement of feeds, delayed administration of parenteral nutrition, and challenges in providing adequate nutrient intake due to practical limitations and increased requirements, such as delayed initiation of Human Milk Fortifiers (HMF), feed intolerance and interruptions, fluid restrictions, inadequate electrolyte and mineral provisions, and the pathophysiology of morbidities. Neonatal Intensive Care Unit (NICU)-related stress and social determinants of health may also exacerbate these challenges [2,4,5,6].

Micro-preterm infants, defined by us as those born at ≤25 + 6 weeks gestation, experience shortened time in utero, which places them at a very high risk of nutrient deficits [7]. These infants often face various other health challenges related to their prematurity, including patent ductus arteriosus (PDA), brain injury, necrotizing enterocolitis (NEC), chronic lung disease (CLD), and retinopathy of prematurity (ROP) [7,8,9,10,11,12,13,14]. Moreover, micro-preterm infants are particularly vulnerable to neurodevelopmental delays and challenges, which can have long-lasting implications for familial dynamics, societal interactions, and healthcare systems [15,16,17,18].

Providing energy, macro- and micro-nutrients within recommended ranges, and enhancing in-hospital growth of very low birthweight (VLBW) infants are critical due to their significant impact on later growth outcomes, morbidity rates, neurodevelopment, and long-term quality of life [1,3,5,19]. In an effort to enhance overall care and optimize outcomes for this vulnerable population, the NICU team at Sunnybrook Health Science Centre (SHSC) implemented a nutrition-care bundle in 2015 in collaboration with the Vermont Oxford Network (VON) [7]. The primary aim of the current study is to delineate and compare growth, the incidence of neonatal morbidities, and long-term outcomes of micro-preterm infants before and after the implementation of the nutrition-care bundle.

## 2. Materials and Methods

This retrospective before-and-after study was conducted at SHSC, a tertiary care unit in Toronto, Ontario, with approval from the Research Ethics Board at Sunnybrook Health Science Centre on October 22, 2019 (Project Identification Number 366-2019).

### 2.1. Intervention

In 2014, the NICU team initiated a quality improvement project aimed at enhancing outcomes for micro-preterm infants. Subsequently, a nutrition-care bundle was implemented encompassing several components: initiation of parenteral nutrition (PN) at birth, introduction of enteral feeds and probiotics within 6 h post-birth, addition of powdered bovine HMF (Similac^®^ Human Milk Fortifier, Abbott Nutrition, Abbott Park, IL, USA; Enfamil^®^ Human Milk Fortifier, Mead Johnson Nutrition, Chicago, IL, USA) at enteral intakes of 120 mL/kg/d, judicious fluid management, infusion of sodium acetate solution in umbilical arterial lines, and maintenance of intravenous lines and PN beyond 120 mL/kg/d of enteral intakes. Implementation of the nutrition-care bundle was accomplished in June 2015. The salient nutrition-care bundle changes are summarized in Table 1.

### 2.2. Study Participants

One of the study’s primary aims was to compare the long-term outcomes of infants before vs. after the implementation of the nutrition-care bundle. Therefore, it was important to ensure infants with complete data related to outcomes were included. Thus, infants born at SHSC at ≤25 + 6 weeks gestation and admitted to the NICU, who survived until at least 18–24 months corrected age (CA) and who attended follow-up clinic assessment at 18–24 CA, were retrospectively selected for inclusion in the study. The historic before-nutrition-care-bundle (BNB) cohort comprised infants born between January 2012 and December 2013, while the after-nutrition-care-bundle (ANB) cohort comprised those born between January 2016 and December 2017. Infants born in 2014 and 2015 were excluded due to the transition in implementing the nutrition bundle. All infants included in the study completed their follow-up assessment in person by December 2019, before the COVID-19 pandemic began. Retrospective data collection commenced in August 2021.

### 2.3. Outcomes

The study’s primary aim was to compare the growth of micro-preterm infants at specific intervals, including Day of Life (DOL) 7, 14, 21, 28, and at 4–8 weeks CA, both before and after the implementation of the nutrition-care bundle. The secondary objective was to assess neurodevelopmental outcomes at 18–24 months CA within the two cohorts. Additionally, compliance with changes in the nutrition-care bundle was evaluated.

### 2.4. Data Collection

Infant and maternal baseline characteristics, as well as relevant neonatal data, including anthropometry, as well as enteral and parenteral nutrition during the NICU stay, were collected retrospectively from computerized database and patient medical records.

### 2.5. Growth

Weight (g) using an electronic scale was documented at birth, daily during NICU stay, and at neonatal follow-up clinic visits. Length measurements were not consistently accurate, and thus, changes in length over time were not assessed. Head circumferences (HC) were documented at birth and neonatal follow-up clinic visits but were not consistently documented in the infants’ hospital chart. In the current paper, changes in weight over time were used as a proxy for growth. When we refer to growth, we are mostly assessing changes in weight over time.

Birthweight, the lowest weight achieved in the first week of life, and the DOL birthweight was regained were recorded. Maximum % weight loss was calculated as follows:

(1) $$\%\;\mathrm{Maximum}\;\mathrm{weight}\;\mathrm{loss}=\frac{\mathrm{Birthweight}\;-\;\mathrm{Lowest}\;\mathrm{weight}\;\mathrm{in}\;\mathrm{week}\;1\;\mathrm{of}\;\mathrm{life}}{\mathrm{Birthweight}}\times 100$$

Weights available at postnatal DOL 7, 14, 21, and 28 were also recorded and used to calculate infants’ growth velocity (GV) using the method described by Patel [20], as follows:

(2) $$\mathrm{GV}=\frac{\left[1000\times \;\ln\;\left(\mathrm{final}\;\mathrm{recorded}\;\mathrm{weight}/\mathrm{Birthweight}\right)\right]}{\left(\mathrm{Postnatal}\;\mathrm{day}\;\mathrm{of}\;\mathrm{final}\;\mathrm{weight}\;\mathrm{recorded}\;-\;\mathrm{Postnatal}\;\mathrm{day}\;1\right)}$$

HC data from birth, as well as weight (g) and HC (cm), documented in the follow-up clinic visits at 4–8 weeks CA were recorded. To calculate changes in weight and HC z-scores from birth, weight and HC z-scores were calculated using the Fenton Preterm Growth Charts [21]. Changes in z-score (weight and HC) were calculated as follows:

Change z-score = z-score at DOL 7, 14, 21, 28 or 4–8 weeks CA − z-score at birth(3)

### 2.6. Morbidities

Significant morbidities were closely monitored in micro-preterm infants. These included PDA (confirmed by echocardiography or initiation of treatment), NEC (≥II Bell staging) [22], late-onset sepsis (positive blood or cerebrospinal fluid culture results at >5 postnatal days), brain injury (echo-dense intraparenchymal lesions, periventricular leukomalacia, porencephalic cysts, or ventriculomegaly with or without intraventricular hemorrhage), severe ROP (confirmed stage ≥3, surgery, or angiogenesis inhibitors) [23], and CLD (oxygen/ventilation support at 36 weeks corrected gestational age, CGA) [24].

### 2.7. Neurodevelopment

Cognitive, language, and motor composite scores were assessed using the Bayley Scales of Infant and Toddler Development, Third Edition (Bayley-III) at 18–24 months’ CA [25], conducted by certified testers in the Neonatal Follow-up Clinic. Children with scores below 85 in Bayley-III domains were further identified, focusing on the diagnosis of Global Developmental Delay (GDD) as per DSM-5 criteria [26]. GDD is characterized by a significant delay (at least two standard deviations below the mean with standardized tests) in at least two developmental domains, including gross or fine motor skills, speech/language, cognition, social/personal skills, and activities of daily living, specifically for children under 5 years old. Information related to visual impairment and hearing loss was also recorded from the follow-up clinic visit records.

Furthermore, all infants in the study were investigated for incidence of cerebral palsy (CP) and its associated GMFCS-E&R scores [27,28], as well as autism spectrum disorder (ASD) based on DSM-5 criteria [26] considering levels of support in Social Communication and Repetitive Restricted Behaviours [26]. The neurological examination was conducted by developmental pediatricians (RB, PTC) to assess motor findings indicative or confirmatory of CP. Infants meeting the criteria for CP were assigned a Gross Motor Functional Classification System-Expanded & Revised (GMFCS-E&R) score. Moreover, all children were screened for ASD. If the screening yielded positive results or if there were concerns regarding ASD, a comprehensive developmental assessment, including the Autism Diagnostic Observation Schedule, was administered (by RB, PTC) to determine whether they met the criteria for ASD.

### 2.8. Statistical Analysis

Early growth trajectories across the two cohorts were visually assessed by plotting weight and weight z-scores across gestational age using the R package (v4.1.1 R Core Team 2021). The effect of the nutritional bundle on early growth trajectories was estimated using mixed effects linear models of weight z-score change from birth and GV at DOL 7, 14, 21, and 28 using the SAS MIXED procedure (SAS Version 9.4). Models were controlled for baseline (birthweight or birthweight z-score) and gestational age at birth, as well as their interaction with DOL, allowing for a waning effect of birth parameters on the growth trajectories across time. Given the large number of multiple births, models also controlled for a random family effect. Similarly, we compared the change in weight and head circumference z-scores at 4–8 weeks CA, controlling for baseline (weight or head circumference z-score at birth), gestational age at birth, and a random family effect. In an exploratory fashion, we assessed the impact of eleven potential confounders (male, multiples, systemic steroids, oxygen, delivery, brain injury, CLD, PDA, late-onset sepsis, NEC, and ROP) on neurodevelopmental outcomes at 18–24 months CA We then compared neurodevelopmental outcomes across the two cohorts using a repeated measures mixed effect model (repeated across three domains), controlling for systemic steroid use, delivery method, and a random family effect. There was no interaction between domain and cohort; therefore, we estimated a single cohort effect across the three domains. An alpha of 0.05 was used for significance for all analyses.

## 3. Results

### 3.1. Maternal–Newborn Characteristics

**Figure 1:**
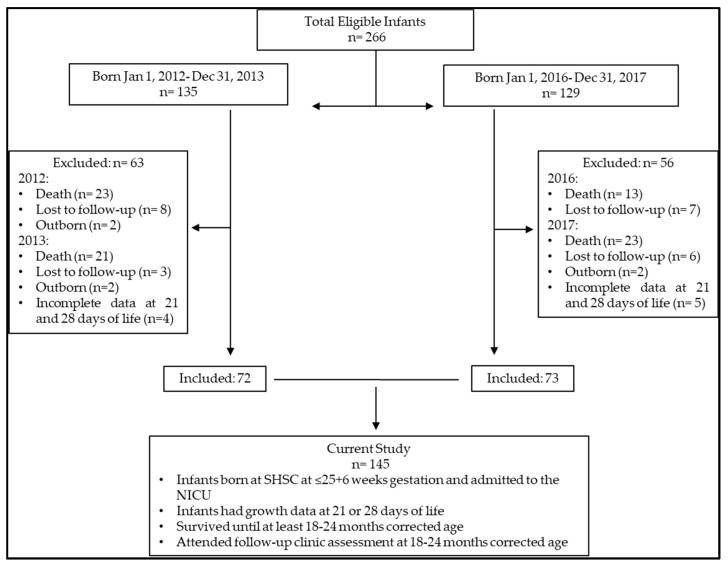
Flow diagram of micro-preterm infants included in the present study.

**Figure 2:**
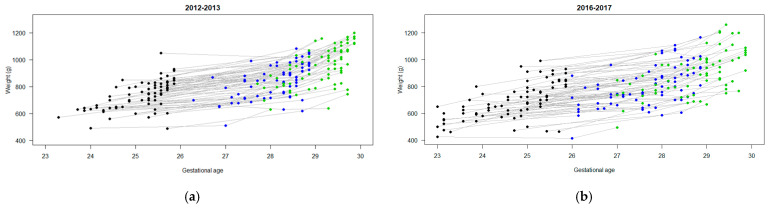
Weights of infants at birth (black dots), day of life 21 (blue dots), and day of life 28 (green dots): (**a**) before nutrition bundle; (**b**) after nutrition bundle.

**Figure 3:**
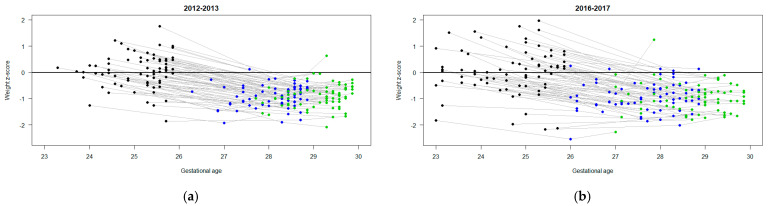
Weight z-score of infants at birth (black dots), day of life 21 (blue dots), and day of life 28 (green dots): (**a**) before nutrition bundle; (**b**) after nutrition bundle.

Of the 266 micro-preterm infants born at SHSC in the two time periods, 145 were included in the final analysis (Figure 1). Maternal and infant characteristics by cohort are outlined in Table 2. Infants had mean ± SD birthweights of 733 g ± 107 g and 694 g ± 141 g, and a median (IQR) gestational age at birth of 25.3 (24.7, 25.6) and 24.9 (24.0, 25.3) weeks in the BNB and ANB periods, respectively. Figure 2 and Figure 3 highlight the differences and variability in weight, weight z-score, and gestational age at birth (and over 4 weeks) between both cohorts. Male sex prevalence was higher (53% vs. 40%), and there was a higher proportion of infants of multiple pregnancies born in the ANB cohort (26% vs. 11%) compared to the BNB cohort (Table 2).

### 3.2. Nutrition and Growth Outcomes

Throughout the study period, the use of the mother’s own milk was promoted. When it was unavailable, banked donor’s milk was fed until infants were 33 weeks CGA, when preterm infant formula was introduced.

Compliance with the nutrition-care bundle was observed in the ANB cohort. All infants in the ANB cohort received the updated PN solution within the first hour after birth; sodium acetate was infused in the arterial line for all infants, and fluid and nutrition orders were based on birthweight in the ANB cohort (Table 3). Time to initiate first feeds, to achieve 120 mL/kg/d enteral feed volume, and to initiate HMF at 22 kcal/oz. and 24 kcal/oz. decreased in the A.N.B. vs. B.N.B. cohorts, respectively. Pasteurized donor breast milk was the predominant first feed provided in the ANB cohort.

Weight z-score changes from birth (Table 3) at DOL 7, 14, 21, 28, and at 4–8 weeks CA were not significantly different between the two cohorts, as was the head circumference z-score change from birth to 4–8 weeks CA At DOL 7; infants in the ANB cohort had a less negative GV compared to the earlier cohort, consistent with descriptive statistics showing lower mean maximum weight loss with ANB infants regaining their birthweight earlier than BNB infants. GV at DOL 14, 21, and 28 was non-significantly different between the two cohorts.

We observed significant interactions between DOL and birth gestational age (*p* < 0.0001) and weight z-score at birth (*p* < 0.0001) on change in weight z-score and between DOL and gestational age (*p* < 0.0001) and weight at birth (*p* = 0.001) on growth velocity (Table 4). Older birth gestational age was associated with significantly less weight z-score and growth velocity loss at DOL 7 but not at DOL 14, 21, and 28. Gestational age did not significantly impact weight z-score change at 4–8 weeks CA but was associated with greater gains/reduced losses in HC z-score. Larger babies at birth (higher birthweight, birthweight z-score, or head circumference z-score) experienced significantly greater losses/fewer gains across all time points.

### 3.3. Morbidities

As outlined in Table 2, more infants in the ANB vs. BNB group were free from neonatal morbidities during their hospital course. Postnatal systemic steroids administration was noted to be higher in the ANB group, while CLD incidence was similar in the ANB and BNB groups, respectively. PDA and PDA ligation were noted to be higher in the BNB cohort (46.5% and 11.4% vs. 24.7% and 4.2%, respectively). Severe R.O.P. was higher in the ANB group.

### 3.4. Neurodevelopment

**Figure 4:**
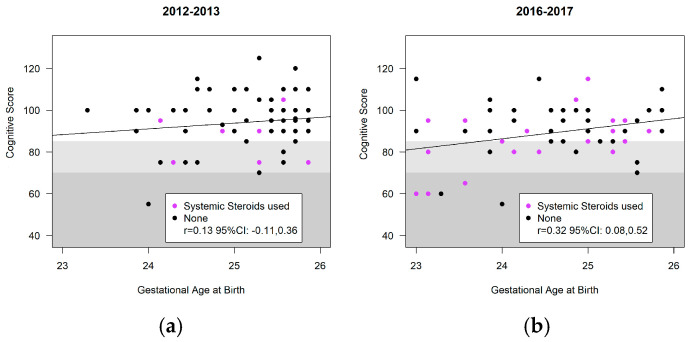
Bayley-III cognitive scores of infants at 18–24 months’ CA: (**a**) before nutrition bundle; (**b**) after nutrition bundle.

**Figure 5:**
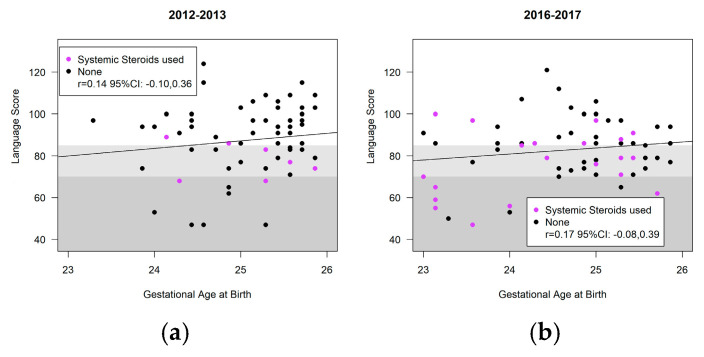
Bayley-III language scores of infants at 18–24 months’ CA: (**a**) before nutrition bundle; (**b**) after nutrition bundle.

**Figure 6:**
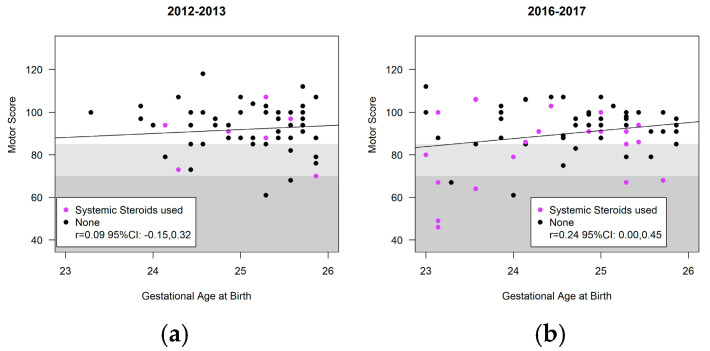
Bayley-III motor scores of infants at 18–24 months’ CA: (**a**) before nutrition bundle; (**b**) after nutrition bundle.

Figure 4, Figure 5 and Figure 6 highlight the variability in Bayley-III scores (cognitive, language, motor) and gestational age at birth between the cohorts. Infants receiving systemic steroids are delineated in the figures. There was no significant interaction between the cohort and the Bayley domains; thus, the cohort effect was estimated across all three domains. There were no significant differences in the composite score of included infants at 18–24 months CA in the ANB vs. BNB cohorts (ANB vs. BNB cohort difference (−1.3, 95%CI [−5.3, 2.7] *p* = 0.53) (Table 5). The use of systemic steroids was associated with significantly lower scores (−8.3, 95%CI [−13.0, −3.5] *p* = 0.0007), and vaginal delivery was associated with significantly greater scores (4.3, 95%CI [0.2, 8.4] *p* = 0.042). The occurrence of CP, GDD, ASD, visual impairment, and hearing loss was notably low in both cohorts. Specifically, within the BNB group, two cases of CP were identified; one presenting with spastic diplegic CP at GMFCS level I–II and the other with spastic quadriplegia at GMFCS level III–IV. In the ANB cohort, one case of spastic quadriplegic CP at GMFCS level IV–V was recorded. Additionally, both cohorts exhibited two cases of visual impairment and four cases of bilateral hearing loss requiring aids. In the ANB cohort, one case met the criteria for GDD, and two cases for ASD with level 3, necessitating very substantial support in both social communication and restricted repetitive behavior, were identified. Similarly, within the BNB cohort, three cases met the criteria for GDD and one case for ASD with level 3, requiring very substantial support in both social communication and restricted repetitive behavior, were reported.

## 4. Discussion

The implementation of a comprehensive nutrition-care bundle in our NICU was initiated through a quality improvement collaboration with VON. The goal of the nutrition-care bundle was to optimize early nutritional intakes, beginning as soon as possible after birth, to promote growth similar to intrauterine growth patterns while minimizing nutrient deficits and associated comorbidities such as BPD, NEC, sepsis, and ROP, ultimately preventing the need for catch-up growth for infants born at or before 25 + 6 weeks gestation [29]. Previous research has established a strong association between early nutritional status and subsequent neurodevelopmental outcomes, with every 10 kcal/kg/d increase in the first week of life associated with a 4.6-point increase in the mental development index score [30].

Despite meticulous planning and implementation, our study did not yield the anticipated improvements in growth and neurodevelopmental outcomes among micro-preterm infants. Infants in the later cohort (born in 2016 and 2017) were noted to be overall smaller at birth compared to infants before the change in nutritional practice (born in 2012 and 2013), with a higher proportion receiving postnatal systemic corticosteroids. Statistical analyses controlled for potential confounders, including differences in birth sizes and systemic steroid use, suggesting that these factors were unlikely to have influenced the lack of significant improvements observed. Of note, survival was higher in the ANB cohort (32.6% deaths in BNB vs. 27.9% in ANB), which is likely contributing to the higher prevalence of early gestational age and/or birth weight in the ANB cohort.

Significant differences in early nutritional practices were observed between the pre-bundle and post-bundle cohorts. The ANB cohort achieved earlier initiation of enteral feeds and reached target feed volumes more expeditiously, reflecting compliance with the nutrition-care bundle and a commitment to optimizing early nutritional support. Although feeds and HMF commenced sooner in the ANB cohort, we did not see increases in NEC as a result, with a pre-bundle NEC rate of 7.1% and 4.2% after. Conversely, we did not see a reduction in rates of late-onset sepsis as would have been expected. Achieving higher feed volume sooner in preterm infants is essential to reduce morbidities related to prolonged use of parenteral nutrition, especially line-related sepsis, increase benefits associated with the use of human milk (provides bioactive and immunomodulatory components such as enzymes, hormones, growth factors, probiotics, oligosaccharides, exosomes, and stem cells), and to allow for earlier enteral nutrient additions to support growth and nutrient retention [31,32,33]. The timing of the addition of the HMF in preterm infants, particularly micro-preterm infants, is controversial. Similar to our findings, recent reviews and meta-analyses have found no safety concerns (such as an increased risk of NEC, feed intolerance, morbidities, or mortality) related to the early addition of HMF, with no significant impact on growth, as a result of earlier addition of HMF [34,35]. Rates of CLD were similar in both cohorts, while PDA and PDA ligation were lower in the ANB cohort despite higher early fluid volumes and lower initial weight loss. This is in contrast to suggestions in the literature regarding increased risk of CLD and PDA with higher early fluid intakes [36,37].

As depicted in Table 3, our results in both cohorts compare favourably to previous studies [4,38], indicating that our NICU’s nutritional care practices were already at a high standard. However, unlike reported improvements in the growth of preterm infants following implementation of nutrition bundles/protocols in some [29,39,40,41,42,43], but not all, studies [44], differences in growth between groups in this study did not persist beyond the early postnatal period. McKinley et al. [39] were able to see improvement in growth in their later epochs after instituting nutrition-care bundles and increasing NICU Registered Dietitians (RD) involvement, with 365 days per year RD coverage. Similarly, Westin et al. [40] reported improved growth and nutrition intakes after the implementation of multiple nutrition guidelines, the use of nutrition software, standard first-day PN, and increased RD employment and education. Rochow et al. [29] instituted nutrition guidelines spanning from birth to 36 weeks CGA, as well as an electronic pre-structured prescription ordering system with a nutrition calculator, which resulted in improved later growth. Highly trained RDs have been part of our clinical team for >30 years and have been providing clinical coverage 365 days per year since 2011. Thus, we likely were not able to ascertain any improvements in growth after the addition of the nutrition-care bundle due to the already instituted nutrition guidelines, first-day standard PN, individualized daily compounding of PN, enhanced macro- and micro-nutrient enteral additives, high use of human milk, preparation of daily feeds in a milk preparation room by trained nutrition technicians, and close monitoring, ongoing focused daily assessments and implementation of infants’ nutrition-care plans by RDs in our NICU year-round. In fact, weight < 10% at hospital discharge for 2012, 2013, 2016, and 2017 in our NICU were 25, 24.2, 24.5, and 22.4%, respectively, which are much lower than the median (IQR) benchmark reported by VON of 52.9 (43.7, 65), 54 (43.5, 66.7), 52.2 (41.7, 65.5), and 52.5 (42.1, 64.7) % for equivalent NICUs during those respective years [45,46,47,48]. Our study suggests that further gains in growth may be limited in NICUs with already established practices and ongoing monitoring by highly trained healthcare professionals, as has been recently shown for overall improvements in mortality and morbidity in VLBW infants [49].

Growth, most commonly weight gain, is a surrogate for assessing the nutritional adequacy of the diet provided to NICU infants. There is a strong association between poor growth and suboptimal neurodevelopmental outcomes of preterm infants. Improving weight gain prior to 40 weeks CGA and from 40–52 weeks CGA have shown the best impact on neurodevelopment [50]. Improving long-term neurodevelopment and quality of life is paramount with the increased survival of micro-preterm infants. Appropriate and balanced intakes of many macro- and micro-nutrients at critical or sensitive periods of brain development are essential to meet the metabolic demands of the growing brain as well as the structural and functional needs of the brain. Optimal nutrient intakes and appropriate growth are modifiable factors in the NICU that have a direct impact on neurodevelopment [19,50]. The observed trends in early weight loss and the timing of regaining the birthweight within the ANB cohort suggest initial benefits conferred by the nutrition-care bundle. However, these gains failed to translate into sustained improvements beyond the early postnatal period. This discrepancy underscores the complex interplay of factors influencing growth and neurodevelopment in micro-preterm infants [2]. Moreover, the higher use of systemic steroids in the later cohort and its association with neurodevelopmental outcomes further underscores the multifactorial nature of neonatal care, a factor not explicitly addressed in previous studies. Additionally, our study’s limitations, including its retrospective nature and relatively small sample size, warrant cautious interpretation of the results and emphasize the need for larger-scale prospective studies to confirm and extend our observations.

Despite these limitations, our study contributes to the growing body of knowledge on neonatal nutrition and highlights the complexity of managing growth and neurodevelopmental outcomes in micro-preterm infants. Moving forward, future research should aim to elucidate the intricate interplay of various clinical and nutritional factors influencing outcomes in this vulnerable population, with a view toward optimizing long-term growth and neurodevelopmental trajectories.

To our knowledge, this is the first study assessing the impact of a nutrition-care bundle on growth and neurodevelopment in micro-preterm infants. This represents a significant advancement in our understanding of how nutrition interventions affect the outcomes of these vulnerable infants, filling a crucial gap in the existing literature. The inclusion of infants born at such extreme prematurity strengthens the validity and relevance of our findings, given their underrepresentation in previous research. However, it is essential to acknowledge the limitations of our study. Conducted retrospectively at a single centre, our findings may not fully generalize to other NICU settings. Another limitation of this study relates to how we have defined growth. We assessed changes in weight z-score, and head circumference, as well as GV relative to birth. We acknowledge that using birth data to describe changes in z-score and GV may not accurately be assessing growth because calculating z-score change or GV from birth includes early postnatal fluid loss, which technically is not a growth phase (T. Fenton, personal communication, 16 March 2023). We felt it was important to use birthweight rather than DOL 7, as recently suggested in some literature [39], to ensure we have no missing or inaccurate data. Due to the high rate of infant transfers to Level II NICU within our healthcare system, we were not able to assess change in z-scores or GV at 36 weeks or once discharged home.

Additionally, challenges in accurately assessing linear growth due to variability in length measurements introduce complexities to our analysis. Moreover, our study’s relatively small sample size may have limited our analyses’ statistical power and increased the potential for type II errors. Despite our efforts to control for confounding variables and thoroughly analyze the data, these limitations must be considered when interpreting our results. Moving forward, larger-scale studies with prospective designs are needed to confirm and build upon our findings, providing a more comprehensive understanding of how nutrition-care bundles impact the outcomes of micro-preterm infants.

## 5. Conclusions

In conclusion, while the introduction of a nutrition-care bundle led to initial improvements in early growth indicators without exacerbating neonatal morbidities, these gains were insufficient to enhance later growth or neurodevelopmental outcomes in our NICU, characterized by close ongoing nutritional care and monitoring. Our study underscores the complexity of nutritional management in micro-preterm infants and highlights the need for tailored interventions addressing their multifaceted needs. Future research should aim to elucidate the intricate interplay of various clinical and nutritional factors influencing outcomes in this vulnerable population, with a view toward optimizing long-term growth and neurodevelopmental trajectories.

## Figures and Tables

**Table 1:** Change in clinical care practice related to the updated nutrition-care bundle.

	Before Nutrition Bundle	After Nutrition Bundle
TFI ordered at birth (mL/kg/d)	80	100
PN ordered at birth	10% dextrose 2.5% amino acids 1.5 mmol/100 mL calcium gluconate	10% dextrose 5% amino acids 2 mmol/100 mL calcium gluconate 2 mmol/100 mL sodium acetate
Volume of PN ordered at birth (mL/kg/d)	80	50
Weight used to order fluids DOL 1–5	Daily weight	Birthweight
Use of complex fluid sheet to consistently include all fluids in actual intakes	No	Yes
Umbilical arterial line solution	Na chloride (7.7 mmol/100 mL)	Na acetate (7.7 mmol/100 mL)
Age enteral feeds initiated after birth	variable	By 6 h
Initiation of probiotics	No	Yes
Feed volume when PN discontinued (mL/kg/d)	120	Beyond 120
Initiation of powdered bovine HMF. (mL/kg/d of enteral feeds)	variable	At 120

Abbreviations: TFI, total fluid intake; PN, parenteral nutrition; DOL, day of life; HMF, human milk fortifier.

**Table 2:** Maternal and newborn characteristics.

Maternal–Newborn Outcomes	2012–2013 n = 72		2016–2017 n = 73	
	**% (n)**		**% (n)**	
Male sex	40.3 (29)		53.4 (39)	
Singleton	26.4 (19)		11.0 (8)	
Maternal P.I.H.	1.4 (1)		9.6 (7)	
Magnesium Sulphate	34.7 (25)		67.1 (49)	
Caesarean delivery	63.9 (46)		65.8 (48)	
G.D.M.				
Insulin	1.4 (1)		2.7 (2)	
Diet	4.2 (3)		2.7 (2)	
Antenatal Steroids				
Partial Course	6.9 (5)		15.1 (11)	
Full Course	73.6 (53)		72.6 (53)	
Size for Gestational Age				
Small	8.3 (6)		9.6 (7)	
Appropriate	90.3 (65)		82.2 (60)	
Large	1.4 (1)		8.2 (6)	
	**median (Q1, Q3)**		**median (Q1, Q3)**	
Gestational age at birth (weeks)	25.3 (24.7, 25.6)		24.9 (24.0, 25.3)	
Apgar Score				
1 min	5 (2, 7)	n = 71	4 (3, 6)	n = 71
5 min	7 (6, 8)	n = 71	7 (6, 8)	n = 70
	**mean (sd)**	**n**	**mean (sd)**	**n**
Birthweight (g)	733 (107)	72	694 (141)	73
Weight z-score	0.0 (0.6)	72	0.0 (0.9)	73
Birth length (cm)	31.9 (1.9)	62	31.5 (2.4)	49
Length z-score	0.0 (0.8)	62	0.0 (1.0)	49
Birth head circumference (cm)	22.7 (1.2)	72	22.0 (1.5)	68
Head circumference z-score	0.1 (0.8)	71	−0.1 (0.9)	68
Maternal age (years)	30.5 (5.6)	72	32.2 (5.8)	71
Neonatal Morbidities and use of Corticosteroids	**% (n)**	**n**	**% (n)**	**n**
Free from any neonatal morbidity	19.4 (14)	72	28.8 (21)	73
Systemic Corticosteroids	11.1 (8)	72	27.8 (20)	72
Brain Injury or I.V.H. or PHVD	14.1 (10)	71	16.4 (12)	73
C.L.D.	41.4 (29)	70	42 (29)	69
P.D.A.	46.4 (33)	71	24.7 (18)	73
L.O.S.	45.7 (32)	70	46.5 (38)	71
N.E.C.	7.1 (5)	70	4.2 (3)	71
R.O.P.	5.7 (4)	70	14.9 (10)	67

Abbreviations: P.I.H., pregnancy-induced hypertension; G.D.M., gestational diabetes; I.V.H., intraventricular hemorrhage; PHVD, posthemorrhagic ventricular dilatation, C.L.D., chronic lung disease; P.D.A., patent ductus arteriosus; L.O.S., late-onset sepsis; N.E.C., necrotizing enterocolitis; R.O.P., retinopathy of prematurity.

**Table 3:** Nutrition and growth outcomes.

	2012–2013 n = 72		2016–2017 n = 73	
First feeds type	**% (n)**		**% (n)**	
EBM	31.9 (23)		5.5 (4)	
DBM	54.2 (39)		83.6 (61)	
EBM/DBM	13.9 (10)		11.0 (8)	
	**median (Q1, Q3)**	**n**	**median (Q1, Q3)**	**n**
DOL 120 feeds	14 (11, 18)	72	11 (10, 13)	70
DOL HMF 1:50	17 (15, 20.5)	71	11 (10, 13.5)	71
DOL HMF 1:25	20 (18, 25)	71	13 (12, 16.2)	72
DOL lowest weight (days)	4 (4, 5)	72	5 (4, 7)	65
	**mean (sd)**	**n**	**mean (sd)**	**n**
Time first PN provided (min)	54.0 (22.9)	63	59.5 (25.1)	53
Time first enteral feed provided (h)	29.8 (20.6)	65	13.1 (11.8)	67
% max weight loss	10.9 (5.6)	72	7.5 (5.8)	66
DOL weight regained (days)	11.8 (5.8)	72	10.2 (6.)	65
Weight (g)				
Birth	733 (107)	72	695 (141)	73
DOL 7	703 (106)	72	693 (137)	67
DOL 14	781 (112)	72	734 (129)	71
DOL 21	844 (123)	70	806 (151)	72
DOL 28	956 (144)	71	893 (162)	69
4–8 weeks CA.				
Weight (kg)	4.4 (0.6)	65	4.5 (0.6)	60
Weight z-score	−0.5 (0.9)	65	−0.6 (1.1)	60
Length (cm)	53.3 (3.3)	45	54.0 (1.9)	40
Length z-score	−1.0 (1.1)	37	−1.3 (1.2)	28
HC (cm)	37.8 (1.1)	61	37.5 (1.2)	59
HC z-score	0.3 (0.8)	61	−0.1 (0.9)	59
Adjusted Estimates *				
	**2012–2013**	**2016–2017**	**Difference**	
	**Estimate (95%CI)**	**Estimate (95%CI)**	**Estimate (95%CI)**	***p***
Weight z-score change				
DOL 7	−0.7 (−0.8, −0.6)	−0.6 (−0.7, −0.5)	0.1 (−0.1, 0.2)	0.072
DOL 14	−0.7 (−0.8, −0.7)	−0.8 (−0.9, −0.8)	−0.1 (−0.2, 0.0)	0.057
DOL 21	−0.9 (−1.0, −0.9)	−1.0 (−1.1, −0.9)	0.0 (−0.2, 0.1)	0.48
DOL 28	−0.9 (−1.0, −0.9)	−1.0 (−1.1, −0.9)	−0.1 (−0.2, 0.0)	0.15
Growth velocity				
DOL 7	−6.2 (−8.2, −4.3)	−0.7 (−2.8, 1.4)	5.6 (2.7, 8.4)	0.0002
DOL 14	5.6 (3.7, 7.6)	4.5 (2.4, 6.6)	−1.1 (−4.0, 1.7)	0.45
DOL 21	7.4 (5.5, 9.4)	7.5 (5.4, 9.6)	0.1 (−2.8, 2.9)	>0.9
DOL 28	10.0 (8.1, 12.0)	9.5 (7.4, 11.6)	−0.6 (−3.4, 2.3)	0.71
4–8 weeks CA.				
Weight z-score change	−0.6 (−0.9, −0.3)	−0.6 (−0.9, −0.3)	0.0 (−0.5, 0.4)	0.89
HC z-score change	0.2 (−0.1, 0.4)	−0.1 (−0.3, 0.2)	−0.2 (−0.6, 0.2)	0.19

Abbreviations: EBM, expressed breast milk; DBM, donor breast milk; DOL, day of life; HMF, human milk fortifier; PN, parenteral nutrition; CA, corrected age; HC, head circumference. * Adjusted for baseline birthweight, birthweight z-score, HC z-score at birth, gestational age at birth, interaction with DOL, and a random family effect.

**Table 4:** Impact of gestational age at birth and birthweight z-score on growth parameters in hospital and after discharge home.

	Gestational Age at Birth		Baseline *	
	Estimate (95%CI)	*p*	Estimate (95%CI)	*p*
Weight z-score change	GA × DOL F(3,392) 8.56, *p* < 0.0001		BW × DOL F(3,392) 10.22, *p* < 0.0001	
DOL 7	0.2 (0.1, 0.2)	<0.0001	−0.37 (−0.44, −0.30)	<0.0001
DOL 14	0.0 (−0.1, 0.1)	>0.9	−0.48 (−0.55, −0.40)	<0.0001
DOL 21	0.0 (−0.1, 0.1)	>0.9	−0.52 (−0.59, −0.45)	<0.0001
DOL 28	0.1 (0.0, 0.1)	0.17	−0.59 (−0.66, −0.52)	<0.0001
Growth velocity	GA × DOL F(3,392) 7.89, *p* < 0.0001		BW × DOL F(3,392) 5.40, *p* = 0.001	
DOL 7	5.4 (3.1, 7.7)	<0.0001	−0.05 (−0.06, −0.03)	<0.0001
DOL 14	0.8 (−1.5, 3.0)	0.50	−0.03 (−0.04, −0.01)	<0.0001
DOL 21	0.7 (−1.6, 3.0)	0.55	−0.02 (−0.03, 0.00)	0.009
DOL 28	1.5 (−0.8, 3.8)	0.21	−0.02 (−0.03, 0.00)	0.016
4–8 weeks CA.				
Weight z-score change	0.0 (−0.3, 0.2)	0.72	−0.5 (−0.8, −0.2)	0.004
HC z-score change	0.3 (0.0, 0.5)	0.026	−0.6 (−0.8, −0.4)	0.0005

Abbreviations: GA, gestational age; DOL, day of life; BW, birthweight; CA, corrected age; HC, head circumference. * All models control for baseline: weight z-score change controls for weight z-score at birth, growth velocity controls for weight at birth, and HC z-score change controls for HC z-score at birth.

**Table 5:** Motor, language, and cognitive Bayley composite scores at 18–24 months corrected age ^1^.

	2012–2013	2016–2017	Difference ^2^	
	Estimate (95%CI)	Estimate (95%CI)	Estimate (95%CI)	*p*
Motor	96.3 (92.2, 100.4)	95.0 (90.8, 99.3)		
Language	90.4 (86.1, 94.6)	89.1 (84.7, 93.5)	−1.3 (−5.3, 2.7)	0.53
Cognitive	97.0 (92.9, 101.0)	95.7 (91.5, 99.9)		

^1^ Estimates are derived from the linear mixed models equation and are shown with systemic steroids fixed at “none” and vaginal delivery. The use of systemic steroids is associated with significantly lower scores: −8.3, 95%CI: −13.0, −3.5, *p* = 0.0007; vaginal delivery is associated with significantly greater scores: 4.3, 95%CI: 0.2, 8.4, *p* = 0.042. ^2^ There was no significant interaction between cohort and Bayley domain; thus, the cohort effect is estimated across all three domains.

## Data Availability

The data presented in this study are available on request from the corresponding author due to privacy and ethical considerations.
